# A de novo variant in the X‐linked gene *CNKSR2* is associated with seizures and mild intellectual disability in a female patient

**DOI:** 10.1002/mgg3.861

**Published:** 2019-08-15

**Authors:** Daniel L. Polla, Harriet R. Saunders, Bert B. A. de Vries, Hans van Bokhoven, Arjan P. M. de Brouwer

**Affiliations:** ^1^ Department of Human Genetics, Donders Institute for Brain, Cognition and Behaviour Radboud University Medical Center Nijmegen The Netherlands; ^2^ CAPES Foundation Ministry of Education of Brazil Brasília Brazil; ^3^ Department of Human Genetics Radboud University Medical Center Nijmegen The Netherlands

**Keywords:** *CNKSR2*, de novo, exome sequencing, intellectual disability, X‐linked

## Abstract

**Background:**

Eight different deletions and point variants of the X‐chromosomal gene *CNKSR2* have been reported in families with males presenting intellectual disability (ID) and epilepsy. Obligate carrier females with a frameshift variant in the N‐terminal protein coding part of *CNKSR2* or with a deletion of the complete gene are not affected. Only for one C‐terminal nonsense variant, two carrier females were mildly affected by seizures without or with mild motor and language delay.

**Methods:**

Exome sequencing was performed in one female child of a Dutch family, presenting seizures, mild ID, facial dysmorphisms, and abnormalities of the extremities. Potential causative variants were validated by Sanger sequencing. X‐chromosome‐inactivation (XCI) analysis was performed by methylation‐sensitive PCR and fragment‐length analysis of the androgen‐receptor CAG repeat polymorphism.

**Results:**

We identified a de novo variant, c.2304G>A (p.(Trp768*)), in the C‐terminal protein coding part of the X‐chromosomal gene *CNKSR2* in a female patient with seizures and mild ID. Sanger sequencing confirmed the presence of this nonsense variant. XCI analysis showed a mild skewing of X inactivation (20:80) in the blood of our patient. Our variant is the second C‐terminal–affecting *CNKSR2* variant described in neurologically affected females.

**Conclusion:**

Our results indicate that *CNKSR2* nonsense variants in the C‐terminal coding part can result in ID with seizures in female variant carriers.

## 1 INTRODUCTION

Intellectual disability (ID) is a common neurodevelopmental disorder, which has an estimated prevalence of approximately 1%–3% in the general population with a female to male ratio of approximately 1.0 to 1.3–1.4 (Leonard & Wen, 2002). ID is characterized by significant limitations in both intellectual functioning and in adaptive behavior with an IQ below 70, and an onset of cognitive impairment in the development period of an individual (DSM‐5, 2013).

X‐linked intellectual disability (XLID) has been estimated to account for approximately 10% of ID in males, with a much lower approximation in females (Lubs, Stevenson, & Schwartz, 2012). The higher prevalence in males is due to the hemizygous state of the X chromosome. Females who carry an X‐linked variant are generally not, or only mildly affected because of X‐chromosome‐inactivation (XCI). XCI is a random transcriptional silencing process, where one of the two X chromosomes is inactivated starting around gastrulation (Morey & Avner, 2011). Positive or negative selection of one of the two cell populations with fixed inactivation of one or the other of the two X chromosomes may subsequently lead to skewing of XCI. Since the nonmutated X chromosome usually has the advantage, females mostly present with no or a milder phenotype due to preferred expression of the normal allele in most if not all tissues. So far, about 100 genes have been reported to be involved with XLID (Hu et al., 2016).

*CNKSR2* is located on chromosome X and comprises 22 exons. At least four alternatively spliced isoforms have been described (Hsu et al., 2006). *CNKSR2* is highly expressed in the brain (Nagase et al., 1998) and is known to play a role in synaptogenesis (Iida, Nishimura, Yao, & Hata, 2002; Lanigan et al., 2003). Depletion of *CNKSR2* in mouse primary hippocampal neurons results in a reduction of the number of dendritic branches, as well as on total length of neurites per neuron (Hu et al., 2016). *CNKSR2* encodes connector enhancer of KSR‐2 (CNK2), a multidomain protein that functions as a spatial modulator of Rac cycling during spine morphogenesis (Lim, Ritt, Zhou, & Morrison, 2014). The N terminus of CNK2 contains a sterile alpha motif (SAM) domain, which is a putative protein interaction module (Schultz, Ponting, Hofmann, & Bork, 1997), a connector enhancer of kinase suppressor of ras (CRIC) domain, and a PDZ domain that is primarily involved in anchoring receptor proteins to the cytoskeleton (Feng & Zhang, 2009). The middle part contains a proline motif that can bind Vilse/ARHGAP39 that functions primarily as a Rac GTPase‐activating protein (Lim et al., 2014; Lundstrom et al., 2004) and a Pleckstrin homology (PH) domain, which possesses multiple functions including the ability to bind inositol phosphates and various proteins (Haslam, Koide, & Hemmings, 1993; Mayer, Ren, Clark, & Baltimore, 1993). The C terminus contains a PDZ‐binding motif (ETHV) that physically interacts with the PDZ domains of the major PSD proteins densin‐180 (Ohtakara et al., 2002), postsynaptic density 95 (PSD‐95), and synaptic scaffold molecule (S‐SCAM) (Yao et al., 1999). The ETHV motif regulates Ras signaling, which controls neuronal proliferation, migration, differentiation, and death (Bumeister, Rosse, Anselmo, Camonis, & White, 2004; Liu et al., 2009; Yao et al., 1999), and synaptogenesis (Iida et al., 2002; Lanigan et al., 2003).

Deletions of *CNKSR2*, either partially or complete, have been reported in a total of seven male patients with ID and epilepsy from five families (Aypar, Wirrell, & Hoppman, 2015; Houge, Rasmussen, & Hovland, 2012; Vaags et al., 2014). In addition, a frameshift variant, p.(Asp152Argfs*8), and two nonsense variants, p.(Arg712*) and a de novo p.(Arg.729*) have been described in three families with affected males (Damiano et al., 2017; Sun, Liu, Xu, Kong, & Wang, 2018; Vaags et al., 2014). In the family with the p.(Arg712*) nonsense variant, two carrier females were also affected, albeit mildly. The mother had isolated febrile seizures and her daughter presented with unspecified mild learning difficulties and seizures, the latter of which disappeared during puberty. Here, we present a female patient with mild ID and seizures, and a de novo variant leading to a stop codon in exon 20 of the 22 exons in *CNKSR2*.

## 2 CLINICAL REPORT

**Figure 1:**
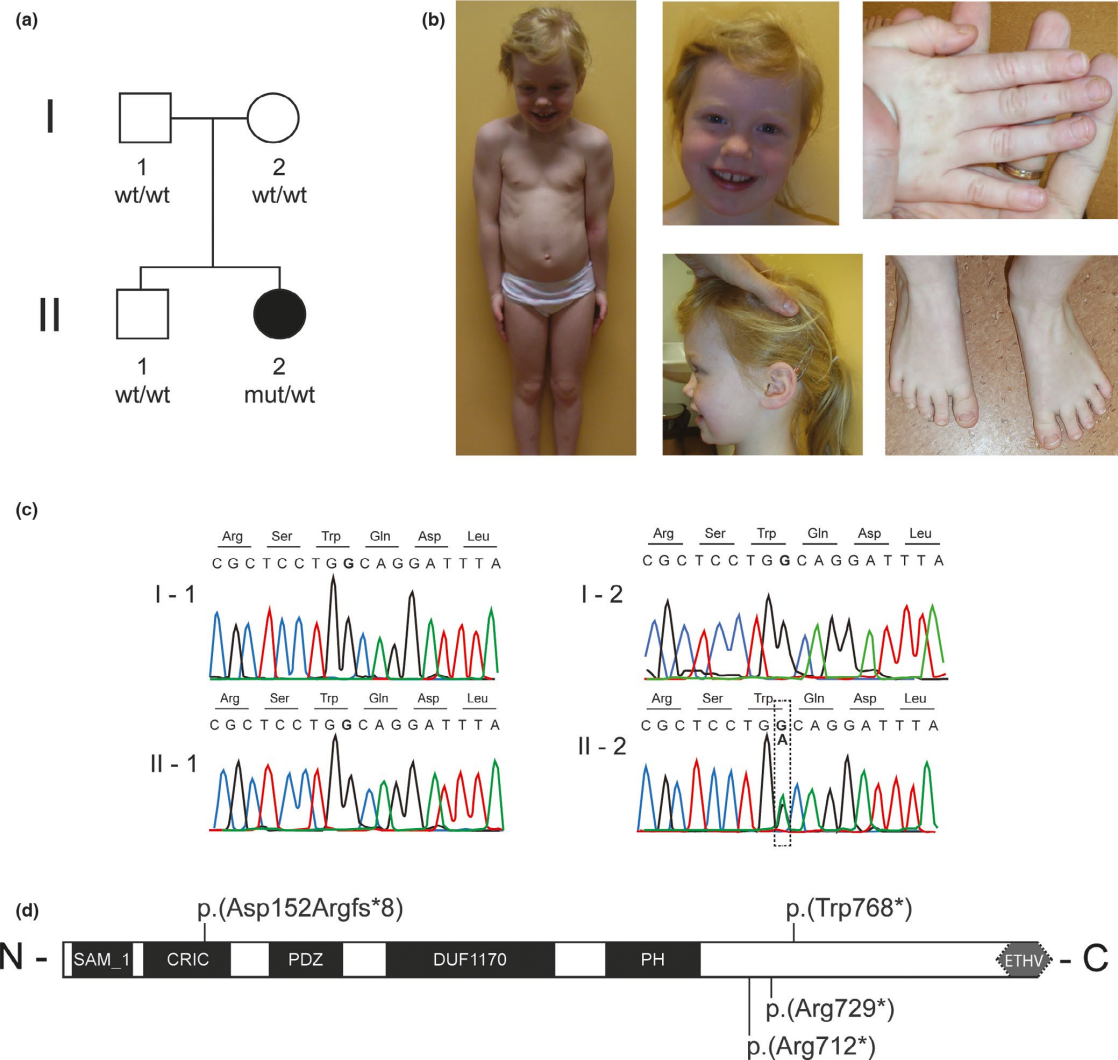
Family ascertained in this study and *CNKSR2* variant. (a) Pedigree of Dutch family W11‐3010. I‐1 = father, I‐2 = mother, II‐1 = brother and II‐2 = affected female patient. Mut = mutated allele; wt = wild type allele. (b) Clinical appearance of affected individual at the age of 7 years, presenting with mild facial dysmorphism including a broad nasal tip and a full lower lip and some distal brachydactyly of the fingers, pes planus, and clinodactyly of the 4th and 5th toes. (c) Electropherograms of the patient and her unaffected family members showing the presence of the variant c.2304G>A (p.(Trp768*)) in the patient only, confirming it to be de novo. The variant is highlighted by a black‐dotted box. The mutated base pair and corresponding amino acid residues are printed in bold. (d) Schematic representation of human CNKSR2 including the positions and the predicted effect of the three identified variants, p.(Asp152Argfs*8) (Vaags et al., 2014), p.(Arg712*) (Damiano et al., 2017), p.(Arg729*) (Sun et al., 2018), and p.(Trp768*) (this report), in *CNKSR2* and on protein level. Black boxes represent the different domains: SAM_1 = sterile alpha motif, CRIC = connector enhancer of kinase suppressor of ras, PDZ = PDZ domain; DUF1170 = domain of unknown function; PH = Pleckstrin homology domain. Gray box represents: ETHV motif = Glu‐Ser/Thr‐Xaa‐Val, or E‐S/T‐X‐V in single letter amino acid code (where Xaa/X is any amino acid residue) motif at the COOH terminus

We ascertained a Dutch family with one female child who represented with ID and seizures (Figure 1a). Written informed consent was obtained and this study was approved by the institutional review board Commissie Mensgebonden Onderzoek Regio Arnhem‐Nijmegen. Pregnancy and delivery after 38 weeks of gestation of the female patient were unremarkable. She had a normal birth weight of 3200 g. Her motor development was delayed, she started walking at 16 months (Table 1). She spoke at the age of 2 years, only a few simple sentences. At the age of 6 years, she has a length of 119 cm (25th centile) and a head circumference of 52.5 cm (80th centile). Her IQ level was tested and she had a total IQ of 71. In addition, the patient suffered from seizures occurring at an onset of 5 years old. At this time of investigation, at the age of 7 years, she presented with mild facial dysmorphism including a broad nasal tip and a full lower lip and some distal brachydactyly of the fingers, pes planus, and clinodactyly of the 4th and 5th toes (Figure 1b). The patient had also nasal speech. MRI of the brain revealed no structural abnormalities. Further genetic testing, including *FMR1* and chromosome analysis using 250K SNP array, showed no abnormalities.

**Table 1:** Clinical descriptions of the patients with *CNKSR2* variants

Reference	Present patient, W11‐3010 II‐2	Damiano et al., 2017	Damiano et al., 2017	Sun et al., 2018	Damiano et al., 2017	Vaags et al., 2014	Houge et al., 2012	Aypar et al., 2015	Vaags et al., 2014	Vaags et al., 2014	Vaags et al., 2014
Ethnicity	**Dutch**	**Ashkenazi**	**Ashkenazi**	Chinese	Ashkenazi	French	NR	NR	Norwegian	French	Canadian
Age(s)	**15**	**16**	**NR (“mother”)**	8	12/18/NR	56/58/62	5	7	8	12/13	6/8
Gender	**Female**	**Female**	**Female**	Male	Three males	Three males	Male	Male	Male	Two males	Two males
mRNA change	**c.2304G>A**	**c.2314C>T**	**c.2314C>T**	c.2185C>T	c.2314C>T	c.453dup	−	−	−	−	−
Protein change	p.(Trp768*)	p.(Arg712*)	p.(Arg712*)	p.(Arg729*)	p.(Arg712*)	p.(Asp152Argfs*8)	Deletion at Xp22.12	Deletion at Xp22.12	Deletion at Xp22.12	Deletion at Xp22.12	Deletion at Xp22.12
Clinical features											
Intellectual disability	**Mild**	**Mild**	−	+	1/1	3/3	Mild/moderate	“Apparent”	+	2/2	1/1
Developmental delay	**+**	**NR**	−	+	2/2	2/2	+	+	NR	NR	1/1
Epilepsy/ Seizures	**+**	**+**	**+**	+	3/3	3/3	+	+	+	1/2	2/2
ADHD	−	−	−	+	2/3	3/3	+	NR	+	2/2	2/2
Language loss	−	**Mild**	−	+	3/3	3/3	−	−	+	2/2	2/2
Sleep disorder	−	**NR**	**NR**	NR	NR	NR	−	NR	+	1/1	1/1
Psychomotor delay	**+**	**Mild**	**NR**	NR	1/1	3/3	+	+	+	2/2	2/2
Additional features	−	−	−	−	−	Minor cortical atrophy (1/3)	Borderline microcephaly	Balance problems	−	−	−

Note

Our patient and the previously reported female with a *CNKSR2* variant are printed in bold.

Abbreviation: NR, not reported.

Exome sequencing was performed on DNA isolated from peripheral blood to identify all potential variants in this patient as described previously (Riazuddin et al., 2017). Selection of the nucleotide variants was performed by using a nine‐tier filtering strategy, specifically designed for de novo analysis or recessive/X‐linked inheritance. The latter was performed as described previously (Riazuddin et al., 2017). For the de novo analysis, we selected variants that are shown when 20%–80% of the aligned reads showed the variant nucleotide. For insertions or deletions (“indels”), we selected those variants with a frequency of 5%–80%. The variant should also be present in less than one percent of the alleles in the ExAC database (Lek et al., 2016) and our in‐house database (Department of Human Genetics, Nijmegen; 12,000 exomes). Furthermore, the variant should be present nonsynonymously in the exon region, or be present in a canonical splice acceptor or donor site. Lastly, we only considered variants that were present in two or more reads and were potentially de novo as they were not present in either parent. This resulted in 16 potentially causative variants, all of which were potentially de novo (Table S1)**.**

Next, we prioritized all of our 16 variants using a five‐tier strategy for potential pathogenic de novo variants only: (a) brain expression of the candidate gene must be greater than or equal to five TPM (transcripts per million) (according to the EST profile: www.ncbi.nlm.nih.gov/unigene), (b) candidate genes should not be deleted more than three times in the Database of Genomic Variants, (c) candidate genes should not be listed as causing non‐ID syndromes in OMIM, (d) candidate genes should have a LoFs pLI≥0.9 or a missense *Z*‐value ≥3.09 (standard deviation from the mean) according to ExAC browser (Lek et al., 2016), and (e) missense variants should have a CADD ≥15 as recommended by the CADD website (http://cadd.gs.washington.edu) (Kircher et al., 2014). The CADD score was not considered for indels, splice site, and nonsense variants. After these steps, four potential causative variants were left.

Sanger sequencing of the four potential causative variants confirmed the presence of only one variant: the potentially pathogenic nonsense variant, c.2304G>A (p.(Trp768*)), in exon 20 of the X‐chromosomal gene *CNKSR2* (NM_014927.3; Figure 1c). This variant was found back neither in the mother nor the father. Analysis of the X‐chromosome‐inactivation status via methylation‐sensitive PCR and fragment‐length analysis of the androgen‐receptor CAG repeat polymorphism, showed a mild skewing of X inactivation (20:80) in the blood of our patient. Primer sequences and PCR conditions for Sanger sequencing and XCI testing are available upon request.

## 3 DISCUSSION

In this case report, we describe a female with mild ID and seizures and a de novo nonsense variant in *CNKSR2*. Deletions of and loss‐of‐function variants in *CNKSR2* (Figure 1d and Figure S1) have previously been described in 14 male patients with ID of varying severity, seizures, language loss, and psychomotor delay (Aypar et al., 2015; Damiano et al., 2017; Houge et al., 2012; Sun et al., 2018; Vaags et al., 2014). In addition, two females have been described with a *CNKSR2* nonsense variant and a mild phenotype consisting of seizures without or with mild motor and language delay, although no formal IQ testing results were presented (Damiano et al., 2017). Our patient confirms that *CNKSR2* variants can result in a neurological phenotype including ID in female variant carriers, even though *CNKSR2* is subject to XCI (Carrel & Willard, 2005).

The two *CNKSR2* nonsense variants found in affected females both result in a premature termination more than 55 bp 5’ from the last exon–exon boundary. Therefore, these variants are expected to result both in reduced protein expression due to nonsense‐mediated mRNA decay (NMD) and in a truncated protein. Interestingly, the five female deletion carriers reported in the literature do not show neurological symptoms (Aypar et al., 2015; Houge et al., 2012; Vaags et al., 2014). Moreover, one of the other four *CNKSR2* loss‐of‐function variants described (Vaags et al., 2014) does not lead to a phenotype in the one obligate female carrier either. This frameshift variant is predicted to result in NMD as well, but at the same time leads to a truncated protein without the PDZ, DUF1170, PH, and C‐terminal ETHV domains. Except for the latter motif, these domains are all still present in the truncated protein resulting from other two *CNKSR2* point variants (Figure 1d). Since NMD usually still results in 15%–58% expression as compared to controls (Coene et al., 2009; Gomez‐Herreros et al., 2014; Wortmann et al., 2012), we speculate that the absence of multiple CNKSR2 domains leads to a more detrimental effect on residual protein function resulting in complete skewing of XCI, and thus to the expression of only the normal allele in specifically brain tissue of female patients. Accordingly, variants that only remove the C‐terminal ETHV motif result in expression of the mutant allele in some brain cells and hence can cause neurological features such as ID, language and motor delay, and epilepsy, in females. This potential skewing of XCI would likely not be visible in blood as *CNKSR2* is specifically expressed in nervous tissue with extremely low expression levels in other cell types such as whole blood (Consortium, 2013; Figure S2), which indicates that CNKSR2 does not have an essential function in these tissues. This is corroborated by the balanced XCI pattern in one of the females with a *CNKSR2* deletion (ratio 56:43) (Houge et al., 2012) and the mild skewing of XCI in our patient (ratio 20:80).

Taken together, we present a female patient with a de novo nonsense variant in *CNKSR2,* causing mild ID and seizures. We speculate that variants removing only the C terminal of CNKSR2 may result in brain expression of a truncated protein with most domains present, which can cause the novel *CNKSR2*‐related features as well as in neurological features such as ID, language and motor delay, and epilepsy, in females.

## CONFLICT OF INTEREST

The authors declare that there is no conflict of interest regarding the publication of this article.
