# Supplementary material for: A de novo variant in the X‐linked gene CNKSR2 is associated with seizures and mild intellectual disability in a female patient
Source: Mol Genet Genomic Med. 2019 Aug 15;7(10):e00861. doi: 10.1002/mgg3.861 (PMC6785448; doi:10.1002/mgg3.861)
Supplement: Supplementary file 1 [file MGG3-7-e00861-s001.docx]

**Supplementary information**

**Fig. S1**. **Schematic overview of the deletions in *CNKSR2***. Schematic overview of the Xq22.12 genomic region including *CNKSR2*. Black bars represent the extent of the deletions. Arrows indicate the position and orientation of all genes in the region. *CNKSR2* is printed in bold. The deletion in the Canadian family was reported to approximate the *RPS6KA3* gene where deletions of which are linked to Coffin–Lowry syndrome. However, the patient was reported not to have the characteristics shown by individuals with Coffin–Lowry syndrome and fine-mapping the breakpoints of the 1.17Mb deletion showed that it is not deleted (Vaags et al., 2014).


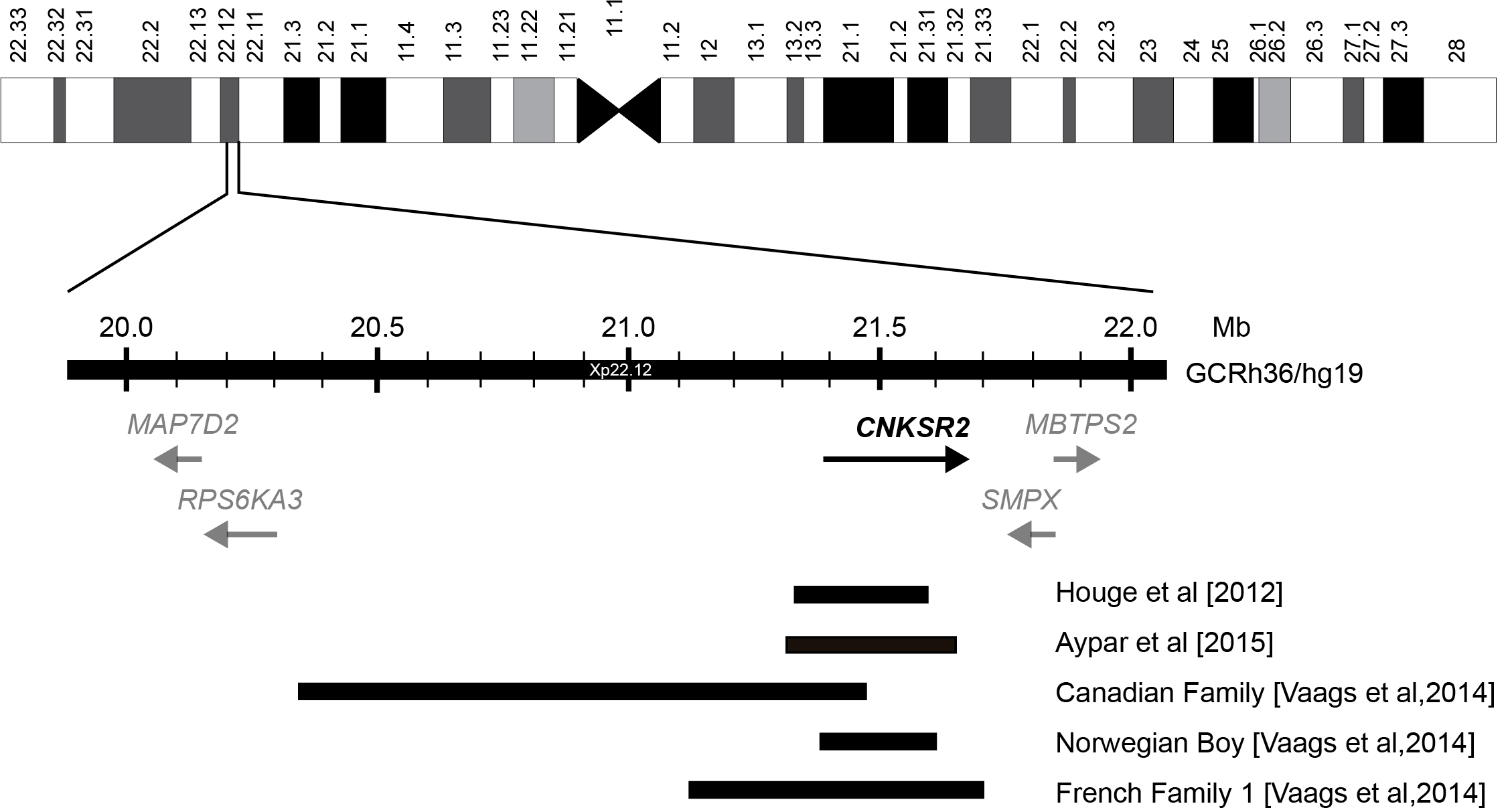


**Fig. S2. Expression of *CNKSR2* in selected human tissues.** *CNKSR2* expression levels as determined by RNA sequencing (Consortium, 2013). Levels are given in transcripts per million (TPM).


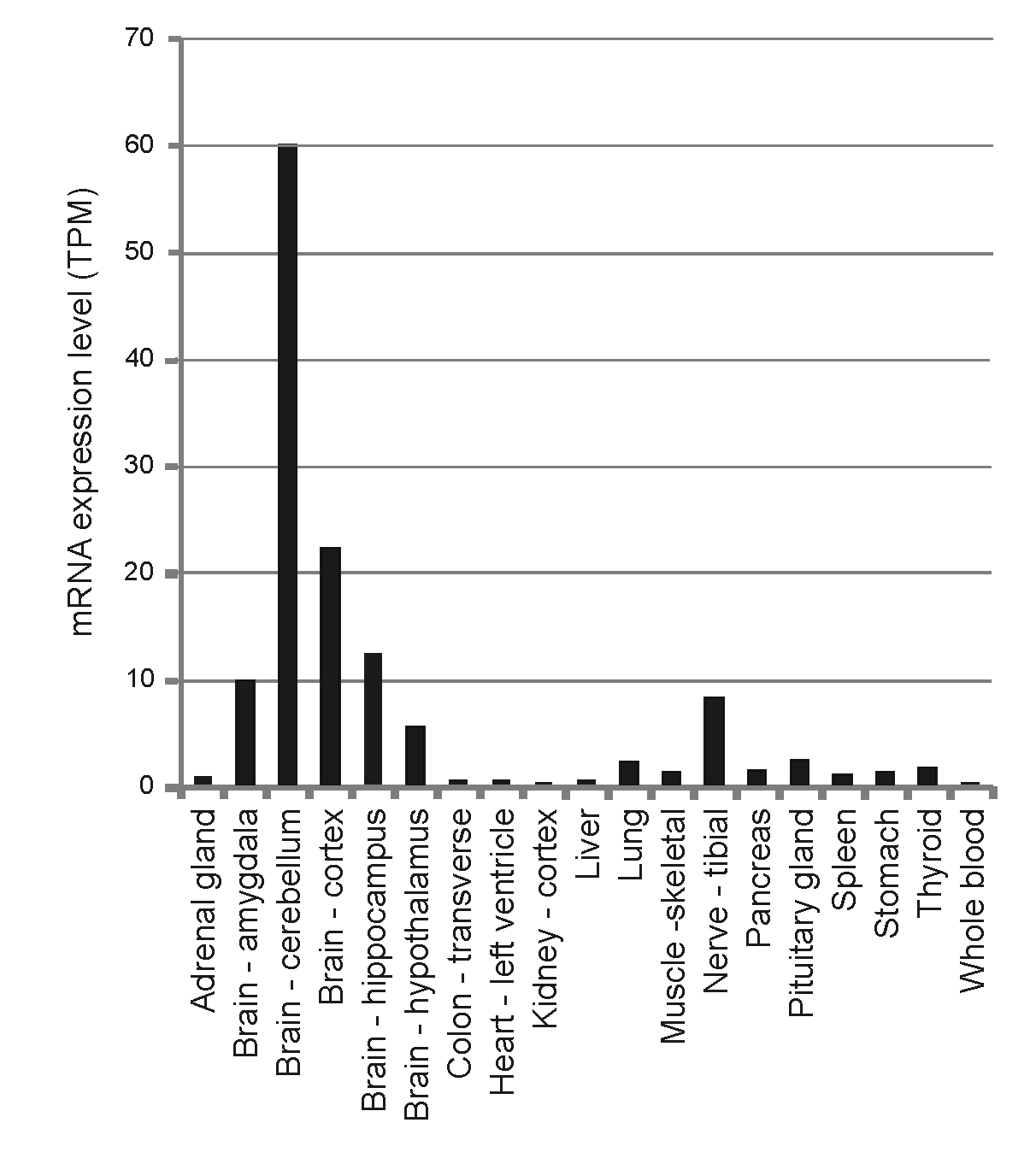


**Table S1. Potentially causative variants after selection of potential pathogenic variants.** Selection of *de novo* and biallelic variants: i) the percentage ratio of variant reads in comparison to the number of reference reads must be between 20-80% and 0-80% for indels or above 80% for homozygous variants, ii) variant frequency must be <1%, iii) variants must be within the exonic regions or canonical splice acceptor or donor site, iv) the identified variant does not lead to a synomynous amino acid change, v) the number of variant reads which are present in the BAM files must be ≥ 2, vi) the variant must not be present in the mother and father in case of the *de novo* assessment. Only variants with a **a**) brain expression ≥5 TPM (transcripts per million), **b**) deleted <4 times in the Database of Genomic Variants (DGV), **c**) not listed as causing a non-ID syndromes in OMIM, **d**) a pLI (probability of LoF intolerance) ≥0.9 or a missense Z-value ≥3.09 (standard deviation from the mean) according to ExAC browser (Lek *et al.*, 2016), and **e**) a LoF variant or a CADD≥15 in case of a missense variant as recommended by CADD (<http://cadd.gs.washington.edu>), were prioritized for validation by Sanger sequencing. Letters in the column ‘prioritization’ indicate which criterium has been met. Chromosomal positions are according to the UCSC Human Genome Browser March 2009, hg19 assembly.

| Gene | Genome | mRNA | Protein | CADD | Prioritization | Confirmed? |
| --- | --- | --- | --- | --- | --- | --- |
| *ADAMTS7* | chr15:g.79058987G>A | NM_014272.3:c.3266C>T | p.(Ala1089Val) | 11 | a,b,c | - |
| *AKAP9* | chr7:g.91718783C>A | NM_005751.4:c.9298C>A | p.(Gln3100Lys) | 17 | a,b,c,e | - |
| *ARMCX4* | chrX:g.100749059G>A | NM_001256155.1:c.5483G>A | p.(Gly1828Glu) | 4 | a,b,c | - |
| *CCDC157* | chr22:g.30768213T>G | NM_001017437.2:c.1273T>G | p.(Trp425Gly) | 14 | a,b,c | - |
| *CNKSR2* | **chrX:g.21627347G>A** | **NM_014927.3:c.2304G>A** | **p.(Trp768*)** | **39** | **a,b,c,d,e** | **Yes** |
| *GNPNAT1* | chr14:g.53248582C>A | NM_198066.3:c.265G>T | p.(Val89Leu) | 21 | a,b,c,e | No |
| *GRASP* | chr12:g.52400862A>C | NM_181711.3:c.59A>C | p.(Asp20Ala) | 18 | a,b,c,d,e | No |
| *HNF1B* | chr17:g.36070591T>G | NM_000458.2:c.1126A>C | p.(Thr376Pro) | 19 | a,b,d,e | - |
| *KMT2D* | chr12:g.49426774T>A | NM_003482.3:c.11714A>T | p.(Gln3905Leu) | 1 | a,b,c,d | - |
| *LRP1* | chr12:g.57605545C>A | NM_002332.2:c.13206C>A | p.(His4402Gln) | 13 | a,c,d | No |
| *SIRT6* | chr19:g.4174802T>G | NM_016539.2:c.880A>C | p.(Thr294Pro) | 0 | a,b,c | - |
| *SIRT6* | chr19:g.4174810C>G | NM_016539.2:c.872G>C | p.(Arg291Pro) | 11 | a,b,c | - |
| *STAG2* | chrX:g.123200052A>T | NM_006603.4:c.2124A>T | p.(Leu708Phe) | 14 | a,b,c,d | - |
| *STARD9* | chr15:g.42985057C>A | NM_020759.2:c.11281C>A | p.(Leu3761Ile) | 22 | a,c,e | No |
| *TIA1* | chr2:g.70456452T>A | NM_022173.2:c.223-2A>T | p.(?) | 8 | a,b,e | No |
| *TNFSF11* | chr13:g.43148519A>G | NM_003701.3:c.80A>G | p.(Glu27Gly) | 17 | b,e | - |
